# Cell Death and Neurodegenerative Diseases: Mechanisms and Cytoprotective Molecules

**DOI:** 10.3390/ijms241411465

**Published:** 2023-07-14

**Authors:** Anne Vejux

**Affiliations:** Team Bio-PeroxIL, “Biochemistry of the Peroxisome, Inflammation and Lipid Metabolism” (EA7270), Université de Bourgogne Franche-Comté, UFR Sciences Vie Terre et Environnement, 21000 Dijon, France; anne.vejux@u-bourgogne.fr

A neurodegenerative disease is a pathological condition affecting neurons, condemning them to death. The term covers a heterogeneous range of disorders affecting different populations of neurons in the nervous system, including the brain and spinal cord. The best-known examples are Alzheimer’s disease, Parkinson’s disease, and multiple sclerosis. Heterogeneity can be seen from both symptomatic and pathophysiological points of view. Variations appear both between different pathologies and between patients within the same pathology. This complicates diagnosis. There is also a very poor correlation between clinical and neuropathological findings. Indeed, the symptoms observed by the clinician do not always correspond to the neuronal affections found after biological analyses such as EMG, EEG, MRI, biopsy analysis, or blood sampling. There are also poor correlations between clinical and genetic findings, as in the case of X-linked adrenoleukodystrophy. Common mechanisms include oxidative stress, apoptosis, and inflammation. The discovery of new processes involved in these pathologies, and of molecules capable of inhibiting them, is at the heart of the latest advances. The findings presented below are based on those described in articles and reviews published in “Cell Death and Neurodegenerative Diseases: Mechanisms and Cytoprotective Molecules”, a Special Issue of *International Journal of Molecular Sciences* (ISSN 1422-0067) and belongs to the section “Molecular Neurobiology”.

In the context of Parkinson’s disease (PD), it has been shown that cytochrome P450 (CYP) isoforms, such as CYP 2D6 or CYP 2E1, may be involved in the development of the disease. In a cell model, inducers of these CYPs prevented identified characteristics of toxicity induced by a parkinsonian neurotoxin: 1-methyl-4-phenylpyridinium (MPP+), for example, decrease in reactive oxygen species (ROS) production, restoration of mitochondrial fusion kinetics, and mitochondrial membrane potential [1].

In Alzheimer’s disease (AD), an important therapeutic target has been identified: remodeling of the GABAergic system. A study was therefore carried out on 3-(5-Methylisoxazol-3-yl)-6-[(1-methyl-1,2,3-triazol-4-yl)methyloxy]-1,2,4-triazolo[3-a]phthalazine (α5IA), an inverse agonist of α5 subunit-containing GABAA receptors (α5GABAARs) with cognition-enhancing properties [2]. Aβ1-42 is able to induce cell loss as well as increased levels of ambient GABA, increased mRNA expression of GABAAR α2,α5,β2/3 subunits and GABAAR R1 and R2 subunits [2]. These changes could contribute significantly to the disruption of inhibitory neurotransmission and normal network activity. Treatment with α5IA restored these parameters [2]. It is well known that Alzheimer’s disease is associated with a progressive loss of neurons due to a process of cell death, but the links between the type of cell death and the different states of the disease are not known. In OXYS rats, a suitable model of the most common (sporadic) form of Alzheimer’s disease, it has been shown that apoptosis is activated at the pre-symptomatic stage. As AD-type pathology progresses, apoptosis and necroptosis are activated as a result of a decline in autophagy-mediated proteostasis [3]. Many clinical, genetic, and pathological features are shared between Alzheimer’s disease and vascular and frontotemporal dementias. This makes diagnosis more than difficult. A study was carried out in patients with Alzheimer’s disease, vascular dementia, and frontotemporal dementia in order to identify the clinical features of these disorders and identify the dysregulated pathways [4]. Specific differences were identified, which could make it possible to distinguish between the dementias. The protein modifications associated with Alzheimer’s disease and their gene expression (amyloid protein precursor and tau protein) after cerebral ischemia, as well as their roles in the post-ischemic period, have been summarized [5]. Thus, deregulation of the precursor genes of the amyloid protein, α-, β- and γ-secretase and tau has been underlined. Reduced expression of the α-secretase gene after cerebral ischemia with recirculation decreases the resistance of neuronal cells to injury. Alzheimer’s disease-related proteins and their genes played a determining role in post-ischemic neurodegeneration and could represent a therapeutic axis.

Abnormalities of myelin, and hence the involvement of oligodendrocytes, are implicated in demyelinating diseases. Such demyelination is found in multiple sclerosis. Cuprizone-induced demyelination is a standard model for multiple sclerosis (MS). Until now, the effects of CPZ on the expression and behavior of microRNAs (miRNAs) have not been studied. Namely, 240 miRNA expression was significantly altered after the treatment of mice with CPZ [6]. Two miRNAs in particular were highlighted: miR-155-5p and miR-20a-5p [6]. Metals have been described as potentially implicated in a number of neurodegenerative diseases, including multiple sclerosis. They can be used as predictive or diagnostic aids, or even as therapeutic targets. Dales & Desplat-Jégo produced a summary of these different possibilities [7].

More generally, molecules such as, for example, the calcium channel blocker nimodipine are described to have neuroprotective activity, but the underlying mechanisms are not known. By using various stress models (osmotic stress, oxidative stress, and heat stress) on different cell types, a decrease in caspase activity has been shown as well as an increase in the phosphorylation of the AKT protein and the CREB protein (cyclic adenosine monophosphate response element-binding protein) after nimodipine treatment under different stress conditions [8]. In neurodegenerative diseases, protein aggregation can be observed. This is why autophagy has been studied. Indeed, when autophagy is deregulated, it is accompanied by the accumulation and deposition of irregular proteins. Melatonin is a neuroendocrine hormone mainly synthesized in the pineal gland and can prevent cell death, reduce inflammation, block calcium channels, etc. [9]. The neuroprotective effects of melatonin against various neurodegenerative diseases have been shown in connection with regulating autophagy. A summary of the available data has been compiled on the potential aetiological involvement of alcohol consumption in the onset or development of the main neurodegenerative diseases [10]. This summary showed that there are still many uncertainties to be resolved before therapeutic solutions can be envisaged.

In addition to research into the mechanisms involved in neurodegenerative diseases, the search for molecules capable of inhibiting toxicity is just as important. In neurodegenerative diseases, such as leukodystrophies, there is an increase in the levels of very long-chain fatty acids (VLCFAs) in blood and tissues. These VLCFAs are toxic to oligodendrocytes [11]. The use of docosahexaenoic acid (DHA) inhibits VLCFA-induced toxicity [11]. Two reviews also considered the potential use of nutraceuticals (e.g., antioxidants) or dietary lipids in the form of animal or vegetable oils or fatty acids [12,13].

## Data Availability

https://www.mdpi.com/journal/ijms/special_issues/Cell_Death_ND (accessed on 30 June 2023).
